# Comment on “Time Trends in Cardiovascular Event Incidence in New‐Onset Type 2 Diabetes: A Population‐Based Cohort Study From Germany”

**DOI:** 10.1111/1753-0407.70130

**Published:** 2025-07-23

**Authors:** Saraswati Sah, Rachana Mehta, Ranjana Sah

**Affiliations:** ^1^ SR Sanjeevani Hospital Kalyanpur Siraha Nepal; ^2^ Clinical Microbiology, RDC Manav Rachna International Institute of Research and Studies Faridabad Haryana India; ^3^ Department of Paediatrics Dr. D. Y. Patil Medical College Hospital and Research Centre, Dr. D. Y. Patil Vidyapeeth (Deemed‐To‐Be‐University) Pimpri, Pune Maharashtra India; ^4^ Department of Public Health Dentistry Dr. D. Y. Patil Dental College Hospital and Research Centre, Dr. D. Y. Patil Vidyapeeth (Deemed‐To‐Be‐University) Pimpri, Pune Maharashtra India

**Keywords:** cardiovascular outcomes, myocardial infarction, population‐based cohort, temporal trends, type 2 diabetes

Dear Editor,

We read with great interest the study by Sarabhai et al., which provides valuable insights into the temporal evolution of cardiovascular events in newly diagnosed type 2 diabetes (T2D) patients in Germany over a 17‐year period [1]. Although the reduction in the 5‐year incidence of coronary heart disease (CHD) and transient ischemic attack (TIA) is encouraging, we believe several methodological and conceptual limitations deserve further discussion to contextualize the findings.

First, the study's exclusion of laboratory parameters such as glycated hemoglobin (HbA1c), lipid panels, and renal function significantly constrains the capacity to adjust for the quality of glycemic and metabolic control—central determinants of cardiovascular outcomes in T2D [2, 3]. Reliance solely on ICD‐10 codes, though validated for primary diagnoses, does not differentiate between stable and unstable angina, nor does it account for silent myocardial infarctions, which are prevalent in diabetic populations [4]. This coding limitation may partially explain the paradoxically unchanged incidence of myocardial infarction (MI) despite improvements in CHD.

Second, the use of chronic obstructive pulmonary disease (COPD) as a proxy for smoking status introduces exposure misclassification. Smoking is a potent modifiable risk factor for both MI and ischemic stroke (IS) [5], and its secular decline in Germany over this period likely contributed to cardiovascular risk reduction. Without direct smoking data, the differential attribution of risk to diabetes‐specific interventions versus population‐wide trends remains unresolved.

Third, the apparent stability in MI and IS incidence may also be an artifact of cohort composition rather than a true epidemiological plateau. Although the authors matched for age and sex, no stratification by socioeconomic status, regional healthcare access, or medication use—particularly statins and antihypertensives—was undertaken. These variables influence the uptake and effectiveness of cardioprotective interventions [6]. Furthermore, the analysis does not disaggregate event timing within the five‐year follow‐up, thereby overlooking early versus late event clustering, which may offer clues about legacy effects from undiagnosed prediabetes.

Fourth, the demographic distribution masks evolving baseline risk. Although mean age and sex proportions remained unchanged, the increased prevalence of hypertension and obesity in the later cohort signals rising baseline cardiovascular risk. Paradoxically, these upward shifts might dilute the observable impact of improved care. Consequently, the unchanged IS and MI rates may reflect counterbalancing effects between therapeutic progress and population‐level risk escalation.

Finally, the study's conclusion that CHD and TIA reduction reflects successful diabetes management may overstate causality. CHD includes chronic, often asymptomatic pathology amenable to long‐term outpatient management and diagnostic advances [7]. In contrast, MI and IS typically reflect acute, irreversible endpoints of vascular injury accumulated over years—possibly even before overt T2D diagnosis [8]. This raises the possibility that prediabetic vascular damage, left unaddressed in this study, may be the principal driver of outcome stagnation.

In conclusion, while the study captures important temporal shifts, a more granular, biomarker‐informed, and longitudinally stratified analysis is essential to disentangle the contributions of diabetes‐specific interventions from broader epidemiological transitions.

## Author Contributions

**Saraswati Sah:** conceptualization, methodology, validation, writing – original draft, writing – review and editing. **Rachana Mehta:** writing – original draft, writing – review and editing. **Ranjana Sah:** supervision, project administration, writing – original draft, writing – review and editing.

## Ethics Statement

The authors have nothing to report.

## Consent

The authors have nothing to report.

## Conflicts of Interest

The authors declare no conflicts of interest.

## Data Availability

The authors have nothing to report, as no data were generated or analyzed in this study.
